# A Cross-Sectional Survey of Healthcare Workers on the Knowledge and Attitudes towards Polio Vaccination in Pakistan

**DOI:** 10.1371/journal.pone.0142485

**Published:** 2015-11-11

**Authors:** Muhammad Umair Khan, Akram Ahmad, Talieha Aqeel, Naila Akbar, Saad Salman, Jawaria Idress

**Affiliations:** 1 Department of Clinical Pharmacy, Faculty of Pharmaceutical Sciences, UCSI University, Kuala Lumpur, Malaysia; 2 Department of Pharmacy Practice, Faculty of Pharmacy, University of Baluchistan, Quetta, Pakistan; 3 Department of Pharmacy, University of Peshawar, Peshawar, Pakistan; 4 Department of Integrated Sciences, Post Graduate Nursing College, Peshawar, Pakistan; Food and Drug Administration, UNITED STATES

## Abstract

**Introduction:**

Pakistan accounts for 85.2% of the total polio cases reported worldwide. Healthcare workers (HCWs) are an integral part of immunization campaigns and source of education for the general public. This study aimed to assess the knowledge and attitudes towards polio vaccination among HCWs providing immunisation and education to general public in Quetta and Peshawar divisions of Pakistan.

**Methods:**

A cross-sectional survey of 490 HCWs was conducted in two major referral public teaching hospitals of Quetta and Peshawar divisions. During February to April, 2015, a random sample of 490 HCWs was invited to participate in this study. Knowledge and attitudes were assessed by using self-administered, anonymous and pretested questionnaire. Descriptive and logistic regression analyses were used to express the results.

**Results:**

A total of 468 participants responded to the questionnaire, giving a response rate of 95.5%. Overall, participants demonstrated good knowledge and positive attitudes towards polio vaccination. The mean knowledge score of HCWs about polio was 13.42±2.39 (based on 18 knowledge questions) while the mean attitude score was 28.75±5.5 (based on 9 attitudes statements). Knowledge gaps were identified about the incubation period of poliovirus (19.5%), management issues (31.9%), use of polio vaccine in mild illnesses (34.7%) and the consequences of the polio virus (36.9%). The majority of participants agreed that all children should be vaccinated for polio (95.1%), while reservations were noted about the need of a booster (38.9%), and sterility issues associated with polio vaccines (43.6%). Internet (n = 167, 37%) and Posters (n = 158, 35%) were the main sources used by HCWs to educate themselves about polio.

**Conclusion:**

Participants in this study had good knowledge and positive attitudes towards polio vaccination. Although the data are indicative of gaps in the knowledge of HCWs, the findings may not be generalized to other hospitals in Pakistan.

## Introduction

Poliomyelitis (polio) is a highly infectious viral disease caused by the polio virus. It is a serious problem in large part of the developing world that is continuously posing threat to the childhood population with critical concerns for social and economic development [1]. The disease is of great public health importance worldwide as it is one of the important causes of disability, especially among children under 5 years of age. Of the 3 strains of wild polio virus (WPV) identified, type 2 WPV has been eradicated globally, while type 1 and type 3 continued to prevail mainly in polio endemic areas. The Polio virus spreads through person-to-person contact, infected faeces, and through the infected secretions from nose and mouth. Polio may remain asymptomatic; however, it may result in partial or full paralysis, respiratory failure, and occasionally, death [2].

In the pre-vaccine era, the prevalence of polio was common worldwide. However, after the introduction of inactivated polio vaccine (IPV) in 1955 and live oral polio vaccine (OPV) in the 1960s, the number of cases declined rapidly in United States, where the last wild poliovirus (WPV) associated polio case was detected in 1991. Since the launch of Global Polio Eradication Initiative (GPEI) in 1988, the incidence of polio cases has been reduced by more than 99%, and the number of countries with endemic polio has been confined from 125 to 3. Presently, polio is endemic in Pakistan, Afghanistan and Nigeria. In 2014, 359 cases of WPV were reported worldwide, of which, 306 cases were reported in Pakistan. In 2015, as of April 29, Pakistan contributed 22 of the 23 WPV cases reported globally [3].

Despite these alarming statistics, Pakistan has made significant improvement towards polio eradication as the number of cases has dropped to 306 in 2014 from more than 20,000 in early 1990s [4]. Still, the country is facing substantial challenges that need to be overcome to eradicate polio completely. In Pakistan, a combination of factors have jeopardised the efforts to get rid of polio including inadequate program management, parental refusal and the opposition of vaccination from the local group [5]. It is reported that 34.7 million children were targeted for polio vaccination in March 2015, of which, 33.6 million children were vaccinated (97%), while remaining children were missed mainly due to security issues (2%) and parental refusal (1%) [6]. The number of polio cases rose significantly in the Federally Administered Tribal Areas (FATA), Khyber Pakhtunkhwa (KPK) and Balochistan province. The number of polio cases in Balochistan increased from 0 in 2013 to 25 in 2014, while in KPK and FATA the numbers have increased from 11 to 68 and 65 to 178 respectively [7]. In Balochistan, the maximum number of cases was reported from Quetta division (19 out of 23). In KPK, Peshawar division is mostly affected by polio, carrying the most burden of the disease (37 out of 68) [4].

Mass polio vaccination campaigns started in Pakistan in April 1994 and are still under way [8]. Rigorous measures have been taken to interrupt the rapid transmission of polio virus in Pakistan in form of supplementary immunization rounds along with the National Immunization Days (NIDs) [8]. Pakistan use trivalent oral polio vaccine (OPV) that contains all the three serotypes in attenuated form [9]. Routine immunization schedule for children in Pakistan includes 4 doses of trivalent OPV at birth, and at ages 6, 10 and 14 weeks. However, on NIDs, 2 doses of OPV are administered at 4 to 6 week intervals for all children aged up to 5 years [9]. Effectiveness of polio vaccine is a major concern in Pakistan. With an average of 8 hours per day of electricity cuts due to load-shedding, maintaining the cold chain may become a concern for the storage of polio vaccines [9].

The healthcare workers (HCWs) are regarded as an important member of the society to combat the endemic diseases like polio. The role of HCWs is exceptional in order to improve access to healthcare and health-seeking behaviour [10]. HCWs are important part of immunization campaigns as they are expected to lead higher vaccination coverage. Furthermore, HCWs are also responsible for ensuring the effectiveness of polio vaccines by adhering to cold chain guidelines [11]. They are the source of information to the parents and the community about the importance and the benefits of vaccination. World Health Organization has emphasised on the need to educate laboratory technicians to prevent the possibility of virus transmission from laboratory to community through contaminated clothing, liquid or air effluents, or improper disposal of infectious materials [12]. Therefore, it is essential for the HCWs to have the correct knowledge and the positive attitudes towards polio vaccination as it is critical for the success of immunization campaigns. Moreover, the evaluation of knowledge and attitudes is an ongoing process that serves as an educational diagnosis among HCWs in order to assess the changing beliefs and behaviour over time [13]. The objective of this study was to assess the knowledge and attitudes of HCWs towards polio vaccination in two major hospitals of Quetta and Peshawar divisions in Pakistan.

## Methods

### Study design, site and participants

A descriptive, cross sectional study was conducted for the period of 3 months from February to April, 2015, among HCWs in Civil Hospital, Quetta, and Lady Reading Hospital, Peshawar, Pakistan. Civil Hospital was selected for this study as this was the major referral teaching hospital of the Quetta division. This hospital is located in Quetta, the capital city of Balochistan. According to census report of 2005, the city had the population of 815,914 and was the 8th most populous city in Pakistan. The Civil Hospital serves the major proportion of Balochistan population as it is a 500 bed multispecialty hospital with different clinical facilities including child health services. Lady Reading Hospital was selected from KPK as it is the oldest and largest teaching tertiary teaching hospital of KPK. It was established in 1924 and is located in Peshawar, the provincial capital of KPK. It has the capacity to accommodate more than 1000 patients. The hospital provides clinical and social services to all the communities in KPK. Both hospitals receive funding from their respective states, which enhances the influx of patients to these hospitals due to their reduced charges. The healthcare workers including physicians, pharmacists, nurses, laboratory technicians and lady health workers were considered eligible to participate in this study.

### Sample size calculation and sampling technique

The sample size was calculated by using Raosoft sample size [14] calculation software in which the population size was kept as 20000, power as 80%, response distribution as 50%, while confidence interval and margin of error was set at 95% and 5% respectively. A sample size of 370 was generated which was adequately powered to estimate the process parameters. However, by anticipating a response rate of 70%, a total of 481 participants were invited to participate in this study. A simple random sampling technique was used to select HCWs (physicians, pharmacists, nurses, laboratory technicians and lady health workers) employed in the selected hospitals by using employees list obtained from both the hospitals.

### Study instrument

The data were collected by a self-administered questionnaire which was distributed to eligible participants for their responses. The data were collected by a team of authors responsible for data collection. The same authors were also responsible for providing explanation to the participants, in case of any request. The questionnaire was pre-tested before distribution to the participants. Initially, a thorough literature review was conducted and the related published papers were short listed for further discussion among authors [15–20]. After comprehensively reviewing the selected papers, a preliminary version of the questionnaire was designed. This questionnaire was then subjected to content and face validity. For content validity, the initial draft was sent to a panel of 3 experts for their opinion on the relativity, simplicity and the importance of the content. The suggested corrections were made to the questionnaire before sending it to a sample of 10 HCWs for their views on making the questionnaire simpler and shorter. The proposed changes were integrated into the questionnaire while ensuring its consistency with the published literature [15–20].

The instrument used in this study was a 46-item structured questionnaire. It was divided in 4 sections. First section investigated the demographic information of participants like gender, age, profession, qualification, marital status, locality and years of experience. Second section included 18 questions which evaluated the knowledge of HCWs about polio. Questions on knowledge were used to assess general knowledge of participants towards polio and its components. It assessed information like causes, sign and symptoms, transmission, incubation period, diagnosis, prevention and its management. The attitudes were evaluated in the third section based on 9 statements. The last section explored the sources of HCWs about polio.

### Data analysis

The responses of participants were analysed by using SPSS v.20. Descriptive analysis was used to express the demographic information in frequencies and percentages. The knowledge questions consisted of Yes/No response categories. Knowledge scores ranged from 0–18 and the cut off level of <12 was set for poor knowledge and ≥12 for good knowledge. The responses of participant over attitudes statement were measured on 4 point Likert scale of agreement. A score of 1 was given to strongly disagree, 2 to disagree, 3 to agree and 4 to strongly disagree. Reverse coding technique was used for negatively worded statements. The scale measured attitude from maximum score of 36 to minimum score of 9. Score of <28 was taken as negative attitude, while ≥28 as positive attitude. Logistic regression analysis was used to assess the association between independent variables (demographic characteristics) and dependent variables (knowledge and attitudes). A p-value of less than 0.05 was reported as statistically significant.

### Ethical approval

The study was ethically approved by Institutional Review Board, Lady Reading Hospital. Moreover, the study was performed as per ethical standard for human experimentation. Written consent was taken from the participants prior the data collection. Participation of respondents was voluntary and their responses were dealt with high level of confidentiality and anonymity. Participants were briefed about the objectives and the significance of research prior to data collection.

## Results

A total of 468 participants responded to the questionnaire, giving a response rate of 97.2%. Majority of participants were male (n = 282, 62.4%), aged 18 to 30 years (n = 257, 56.9%), and had undergraduate degree (n = 210, 46.5%). Physicians (n = 164, 36.3%), nurses (n = 139, 30.8%) and lady health workers (n = 65, 14.3%) were the major respondents in this study. The number of married respondents (n = 351, 77.7%) were higher than the single participants (n = 101, 22.3%). Not much difference was observed between the number of respondents from Quetta (n = 212, 46.9%) and Peshawar (n = 240, 53.1%) (Table 1).

**Table 1:** Demographic information of the participants.

Variables	Number	Percent (%)
Age		
18–30	257	56.9
31–40	65	14.4
41–50	107	23.7
51–60	14	3.1
>60	9	2.0
Gender		
Female	170	37.6
Male	282	62.4
Qualification		
Diploma	190	42.0
Bachelors	210	46.5
Masters	14	3.1
Doctorate	38	8.4
Profession		
Physician	164	36.3
Pharmacist	37	8.2
Nurse	139	30.8
Lab technician	47	10.4
Lady health worker	65	14.3
Experience		
<3	205	45.4
3–6	1	0.2
7–10	104	23.0
>10	142	31.4
Marital status		
Single	101	22.3
Married	351	77.7
Locality		
Quetta	212	46.9
Peshawar	240	53.1

The mean knowledge score of HCWs about polio was 13.42±2.39 (based on 18 knowledge questions). Overall, 71.7% (n = 324) participants exhibited good knowledge of polio. Almost all participants (n = 451, 99.8%) were aware of polio. 96.2% participants (n = 435) correctly answered that polio is a viral disease. Participants were highly knowledgeable about the role of immunization in the prevention of polio (n = 429, 94.9%). Majority participants knew about age group at risk of polio (n = 92.7%, n = 419) and the importance of reporting polio cases to the local authorities (n = 397, 87.8%). On the contrary, HCWs were least knowledgeable about the incubation period of poliovirus (n = 88, 19.5%). Similarly, 68.1% (n = 308) HCWs incorrectly answered that polio is curable. Moreover, 65.3% HCWs (n = 295) incorrectly answered that polio drops should not be given to children with mild illnesses. More than one third of participants (n = 167, 36.9%) incorrectly responded that polio is not a fatal disease. The description of knowledge of participants about polio is summarized in Table 2.

**Table 2:** Knowledge of participants towards polio.

Questions	Correct answers (%)	Incorrect answers (%)
Awareness of polio	451 (99.8)	1 (0.2)
Polio is a viral disease	435 (96.2%)	17 (3.8%)
Polio is also called infantile paralysis as it mostly affects children under 5 years of age	419 (92.7)	33 (7.3)
Sub-clinical symptoms of polio	379 (83.8)	73 (16.2)
Most persons do not develop symptoms	336 (74.3)	116 (25.7)
Incubation period of paralytic poliovirus is 3–6 days.	88 (19.5)	364 (80.5)
Lack of immunization against polio is a major risk factor of polio	365 (80.8)	87 (19.2)
Travel to an area that has experienced a polio outbreak is also a risk factor for children	379 (83.8)	73 (16.2)
Polio is spread by the fecal-oral and respiratory routes	380 (84.1)	72 (15.9)
Polio can also be transmitted through from contaminated food, water and faeces	368 (81.4)	84 (18.6)
Polio is curable	144 (31.9)	308 (68.1)
Patient should be promptly isolated to upon diagnosis to avoid transmission	334 (73.9)	118 (26.1)
Respiratory swab is highly recommended for isolating polio causing organism in suspected patients	367 (81.2)	85 (18.8)
Reporting of suspected paralytic polio has been classified as immediately notifiable and extremely important	397 (87.8)	55 (12.2)
Immunization is the most effective way of preventing polio	429 (94.9)	23 (5.1)
Polio drops should not be given to children in mild illness	157 (34.7)	295 (65.3)
Polio can cause of death of the patient	285 (63.1)	167 (36.9)
Post-polio syndrome is a complication that develops in patient usually 30 years or more after they are first infected	353 (78.1)	99 (21.9)

Note: Knowledge was assessed by giving a score of 1 to correct answer and 0 to wrong answer. The scale measured knowledge from maximum 18 to minimum 0. Scores of <12 were taken as poor knowledge, while a score of ≥12 as good knowledge of Polio. Mean knowledge score was 13.42 ± 1.98.

The results showed that participants with doctorate degree had higher knowledge of polio than the diploma holders (OR = 2.88, p<0.05). The knowledge of physicians were significantly higher than the lady health workers (OR = 2.86, p<0.05). Similarly, participants with more than 10 years of experience were more knowledgeable than participants with less than 3 years of experience (OR = 3.86, p<0.05). The association of the demographic characteristics and knowledge of the participants is expressed in Table 3.

**Table 3:** Correlates of knowledge on polio with demographic variables among healthcare workers Peshawar and Quetta.

Variables	Knowledge on Polio		OR (95% CI)	
	Poor	Good	Crude	Adjusted*
Age				
<30	23.7	76.3	Ref	Ref
31–40	47.7	52.3	0.94 (0.21–1.61)	1.17 (0.37–2.63)
41–50	19.6	80.4	1.48 (0.28–2.42)	1.35 (0.27–2.94)
51–60	22.5	77.5	1.14 (0.39–2.44)	1.68 (0.56–3.17)
>60	11.1	88.9	1.55 (0.42–2.51)	1.48 (0.12–2.92)
Gender				
Female	49.4	50.6	Ref	Ref
Male	15.6	84.4	1.28 (0.46–2.67)	1.41 (0.45–2.78)
Qualification				
Diploma	58.4	41.6	Ref	Ref
Bachelors	14.3	85.7	2.28 (1.11–3.62)^^^	2.49 (1.23–4.52)^^^
Masters	7.1	92.9	2.76 (1.23–4.18)^^^	2.26 (0.78–4.38)^^^
Doctorate	0	100	3.12 (1.46–6.41)^^^	2.88 (0.95–5.21)^^^
Profession				
Physician	6.1	93.9	3.12 (1.44–5.75)^^^	2.86 (0.66–5.26)^^^
Pharmacist	25.4	74.6	2.72 (0.72–4.57)^^^	1.81 (0.65–2.51)
Nurse	48.2	51.8	1.14 (0.14–2.24)	1.24 (0.28–2.46)
Lab technician	73.4	26.6	0.74 (0.21–1.24)	0.78 (0.19–1.35)
Lady health worker	68	32	Ref	Ref
Experience				
<3	19	81	Ref	Ref
3–6	13.8	86.2	1.46 (0.68–3.27)	1.31 (0.16–3.1)
7–10	16	84	1.32 (0.19–2.54)	1.16 (0.38–2.39)
>10	1.7	98.3	4.25 (1.46–7.58)^^^	3.86 (1.27–6.72)^^^
Marital status				
Single	37.6	62.4	Ref	Ref
Married	25.6	74.4	1.27 (0.62–2.73)	1.17 (0.38–2.26)
Locality				
Quetta	55.2	44.8	Ref	Ref
Peshawar	41.6	58.4	1.28 (0.21–1.97)	1.12 (0.14–1.82)

* Adjusted for age, gender, qualification, profession, experience, marital status, locality,

^^^ Significant (p<0.05),

Overall predictive accuracy of the model is 85% Omnibus tests of model coefficients: Chi-square value = 265.840, p<0.001–2 Log Likelihood = 272.887, Nagelkerke R square = 0.639

The mean attitude score of HCWs towards polio vaccination was 28.75 ± 5.5 (based on 9 attitude statements). Overall, 57.7% (n = 261) participants showed positive attitudes regarding polio. A large proportion of respondents (n = 424, 93.8%) strongly agreed or agreed that polio is a very serious illness. Majority of respondents believed that all children should be vaccinated for polio (n = 430, 95.1%). HCWs agreed that polio vaccines should be appropriately stored to ensure its effectiveness (n = 397, 87.8%). However, 56.4% (n = 255) participants believed that polio vaccines have sterility issues. Similarly, 61.1% (n = 276) HCWs responded that polio vaccination should not be repeated as it results in over dosage and cause harm to children. Furthermore, more than one third of participants (n = 161, 35.6%) expressed their disagreement with the statement that the problem of polio is very severe in their region. The description of attitudes of the participants about polio is presented in Table 4.

**Table 4:** Attitudes of participants towards polio vaccination.

Statements	Participants’ responses N (%)			
	Strongly disagree	Disagree	Agree	Strongly agree
Polio is a very serious disease	5 (1.1)	22 (4.9)	119 (26.3)	305 (67.5)
The problem of polio is very severe in your region	68 (15)	93 (20.6)	61 (13.5)	230 (50.9)
Polio vaccines are not capable to reduce the transmission of infection	120 (26.5)	93 (20.6)	49 (10.8)	190 (42)
Polio vaccination should not be repeated as it results in over dosage and cause harm to children	105 (23.2)	71 (15.7)	65 (14.4)	211 (46.7)
Polio vaccines should be appropriately stored in order to be effective	15 (3.3)	40 (8.8)	203 (44.9)	194 (42.9)
Polio vaccines have some sterility issues	116 (25.7)	81 (17.9)	60 (13.3)	195 (43.1)
All children should be vaccinated for polio	7 (1.5)	15 (3.3)	61 (13.5)	369 (81.6)
Communities should actively participate in controlling polio in Pakistan	5 (1.1)	16 (3.5)	89 (19.7)	342 (75.7)
People with polio are less productive than non-disabled ones.	36 (8)	61 (13.5)	150 (33.2)	205 (45.4)

Note: Attitude was assessed by giving a score 1 to strongly disagree, 2 to disagree, 3 to agree, 4 to strongly agree. The scale measured attitude from maximum score of 36 to minimum score of 9. Score of < 28 were taken as negative attitude, while a score of ≥ 28 as positive attitudes. Mean attitude score was 28.75 ± 5.5

**Fig 1:**
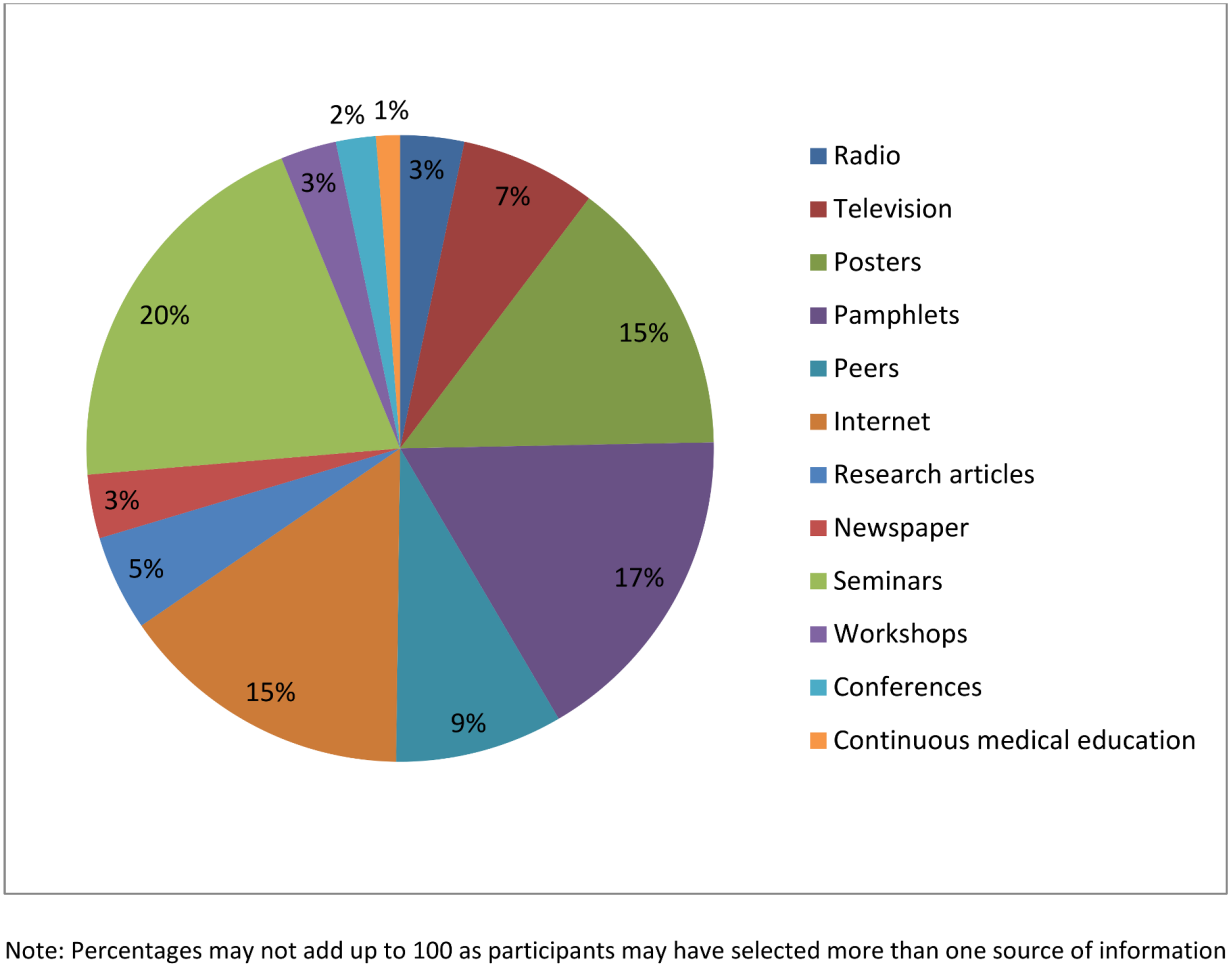
Healthcare workers sources of information about polio.

The participants aged more than 60 years were more likely to have positive attitudes towards polio than participants with 18 to 30 years of age (OR = 3.26, p<0.05). Higher qualification of HCWs was also associated with their positive attitudes. Doctorate degree holders were 4.89 times more likely to have a positive attitudes regarding polio than diploma holders (p<0.05). Physicians’ attitudes were more positive than lady health workers (OR = 2.79, p<0.05), while attitudes of lab technician were significantly poorer than lady health workers (OR = 0.22, p<0.05). Marital status of HCWs also appeared to be a significant predictor of their attitudes as married participants showed positive attitudes towards polio than HCWs who were single (OR = 2.81, p<0.05) (Table 5). Internet (n = 167, 37%) and Posters (158, 35%) were the major sources of information about polio reported by HCWs as shown Fig 1.

**Table 5:** Correlates of attitudes towards polio vaccination with demographic variables among healthcare workers Peshawar and Quetta.

Variables	Attitudes towards Polio vaccination (%)		OR (95% CI)	
	Negative	Positive	Crude	Adjusted*
Age				
<30	28.8	71.2	Ref	Ref
31–40	95.4	4.6	1.34 (0.19–0.60)	1.07 (0.45–2.52)
41–50	36.4	63.6	1.79 (0.47–1.33)	1.03 (0.41–2.61)
51–60	100	0	1.56 (0.18–1.73)	2.08 (0.24–4.8)^^^
>60	22.2	57.7	2.49 (0.3–4.39)^^^	3.26 (0.5–7.38)^^^
Gender				
Female	35.3	64.7	Ref	Ref
Male	28.4	71.6	1.28 (3.4–2.24)	1.32 (1.39–2.93)
Qualification				
Diploma	70	30	Ref	Ref
Bachelors	12.9	87.1	4.26 (1.5–10.3)^^^	4.03 (1.86–9.5)^^^
Masters	100	0	4.43 (1.83–8.1)^^^	4.29 (1.36–10.7)^^^
Doctorate	44.7	55.3	5.4 (1.1–12.4)^^^	4.89 (1.64–13.7)^^^
Profession				
Physician	18.3	81.7	2.7 (1.3–4.41)^^^	2.79 (1.64–4.83)^^^
Pharmacist	64.9	35.1	1.1 (0.32–2.4)	1.73 (0.3–4.48)
Nurse	54	46	1.28 (0.62–2.63)	1.38 (0.12–3.22)
Lab technician	44.8	55.2	0.35 (0.08–0.91)^^^	0.22 (0.09–0.94)^^^
Lady health worker	92	8	Ref	Ref
Experience				
<3	26.3	73.7	Ref	Ref
3–6	100	0	1.2 (0.12–2.18)	1.45 (0.47–2.78)
7–10	74	26	1.32 (0.19–2.54)	1.64 (0.24–3.12)
>10	41.5	58.5	4.25 (1.12–9.43)^^^	3.57 (1.05–7.68)^^^
Marital status				
Single	53.5	46.5	Ref	Ref
Married	39	61	2.74 (1.09–4.79)^^^	2.81 (1.31–5.46)^^^
Locality				
Quetta	20.1	79.9	Ref	Ref
Peshawar	13.8	86.2	1.81 (0.61–2.71)^^^	1.53 (0.58–2.93)

* Adjusted for age, gender, qualification, profession, experience, marital status, locality,

^^^ Significant (p<0.05),

Overall predictive accuracy of the model is 78.2%, Omnibus tests of model coefficients: Chi-square value = 257.852, p<0.001–2 Log Likelihood = 258.982, Nagelkerke R square = 0.428

## Discussion

To the best of our knowledge, the findings reported in this study give a first insight regarding the knowledge and attitudes of HCWs about polio in the 2 most affected regions (Quetta and Peshawar divisions) of Pakistan.

Overall, HCWs exhibited good knowledge about polio. HCWs knew about the polio virus, importance of immunization and the age group most susceptible to the disease. Another survey in Pakistan reported inadequate knowledge among healthcare providers regarding immunization [21]. The likely reason of this discrepancy could be the fact that latter study was conducted more than a decade ago, while the immunization campaigns have been intensified in Pakistan, which may have increased the knowledge of HCWs about polio. The current study also explored areas where knowledge gaps were identified such as incubation period of virus (19.5%), possibility to cure polio (31.9%), administering of polio drops in patient with mild illness (34.7%) and the possibility of death with polio (63.1%). These findings may have far reaching implications as it could be a contributing factor towards the failure of immunization campaigns. It has been reported that health workers are the major source of information for general public regarding polio in Pakistan [22]. Inadequate knowledge of HCWs on important issues like administration of polio vaccines and the consequences of polio may result in transfer of false information. Therefore, the findings suggest the need to customize interventions that explicitly increase HCWs knowledge in the above identified areas. The research findings revealed that the knowledge of lady health workers was relatively lower than pharmacists, nurses and physicians. These results are of great concerns as lady health workers are an important workforce in the anti-polio immunization campaigns in rural and urban slum areas of Pakistan. Eligibility criteria to be a lady health worker include a minimum of 8 years of secondary school education, followed by 15 months training in the prevention and treatment of common illnesses [23]. First, we suggest that the eligibility criteria should be revised to matriculation (10 years of schooling). This would ensure that the lady health workers are exposed to biology related subjects in their schooling, which will form the basis to receive a formal training in health related areas. Second, in addition to their usual training about common diseases, these health workers should also be given special training to manage polio as their role is vital in eradicating polio from the country. External evaluation of lady health workers also indicated the room for improvement in the training program of these workers [24].

The current study also sheds light on the attitudes of HCWs towards polio vaccination. The results are encouraging as HCWs showed positive attitudes towards the storage of polio vaccines. It has been reported on numerous occasions that cold chain maintenance is essential to maintain the effectiveness of polio vaccines [25, 26]. The positive attitudes of health care professionals are vital for the successful cold chain management of polio vaccines [27]. In contrast, the attitudes of HCWs towards immunization were negative elsewhere [27, 28]. The likely reason for this discrepancy could be the recent intensification of polio education program in Pakistan. However, it is important to assess the practices of HCWs as severe electrical load shedding (several hours of power cut a day) is a main hurdle in the cold chain management of polio vaccines in Pakistan [29]. The findings suggest negative attitudes of the participants towards the repetition of polio vaccines and sterility issues. The results are remarkable in a way that HCWs are the main source of information to the public regarding polio [22]. These findings may explain the widely prevalent misconceptions about polio vaccines in Pakistan [30]. The results of multivariate analysis showed that age, qualification, profession, experience and marital status of participants were significantly associated with their attitudes towards polio vaccination. Since no studies were found to relate these findings, we would like to draw researchers’ attention toward this matter to further validate the findings of this study.

The strength of this study is that it highlights an area where not much literature is available. Similarly, inclusion of HCWs from the major referral hospitals of areas highly affected by polio is an additionally strength of this study. However, it is important to mention that the results of this study should be interpreted in the context of potential identified limitations. First, in view of the cross-sectional design, the study is only able to predict the general association between predictor and dependent variables, and should not be taken as cause-and-effect relationship. Second, although the study was conducted in major hospitals of Peshawar and Quetta, the findings may not be generalizable to other hospitals in Pakistan. Third, as a general limitation to knowledge and attitude research, we cannot ignore the tendency of participants to provide more socially desirable responses. Despite the limitations identified, our findings have important implications for the prevention of polio and future research.

The findings of this study could have significant implications for the development of future strategies for polio education and provision of information to the HCWs to improve their knowledge and attitudes towards polio vaccination as they are the important member of anti-polio campaigns, and important source of information for the general public. Additionally, this study highlights the need for further research in areas such as practices of HCWs towards cold chain management of polio vaccines and inclusion of hospitals from other regions of Pakistan.

## Conclusion

Overall, HCWs exhibited good knowledge and positive attitudes towards polio vaccination. However, there is still room for improvement in certain areas like management of polio and use of polio vaccines in children with mild illnesses. The attitudes of respondents were negative about the gravity of the polio crises in their respective regions, repetition of polio vaccines, and sterility issues associated with polio vaccines. These shortcomings are potentially important as they may explain the failure of anti-polio campaigns despite the consistent efforts by the global community over the past few decades. Since the study was conducted in 2 hospitals, the findings may not be generalizable to other hospital settings; hence, further studies need to be conducted to validate the findings of this study.
